# Optically-Thin Broadband Graphene-Membrane Photodetector

**DOI:** 10.3390/nano10030407

**Published:** 2020-02-25

**Authors:** Tania Moein, Darius Gailevičius, Tomas Katkus, Soon Hock Ng, Stefan Lundgaard, David J. Moss, Hamza Kurt, Vygantas Mizeikis, Kȩstutis Staliūnas, Mangirdas Malinauskas, Saulius Juodkazis

**Affiliations:** 1 Optical Sciences Centre, Swinburne University of Technology, John St., Hawthorn, VIC 3122, Australia; tkatkus@swin.edu.au (T.K.); saulius.juodkazis@gmail.com (S.J.); 2 The ARC Training Centre in Surface Engineering for Advanced Materials (SEAM), School of Science, Swinburne University of Technology, Hawthorn, VIC 3122, Australia; 3 Laser Research Center, Faculty of Physics, Vilnius University, Saulėtekio Ave. 10, LT-10223 Vilnius, Lithuania; 4 Department of Electrical and Electronics Engineering, TOBB University of Economics and Technology, Ankara 06560, Turkey; 5 Research Institute of Electronics, Shizuoka University, 3-5-1 Johoku, Naka-ku, Hamamatsu 432-8561, Japan; 6 Dep. de Física, Universitat Politècnica de Catalunya (UPC), Colom 11, E-08222 Terrassa, Spain; 7 Institució Catalana de Recerca i Estudis Avançats (ICREA), Passeig Lluís Companys 23, E-08010 Barcelona, Spain; 8 Tokyo Tech World Research Hub Initiative (WRHI), School of Materials and Chemical Technology, Tokyo Institute of Technology, 2-12-1, Ookayama, Meguro-ku, Tokyo 152-8550, Japan

**Keywords:** optically thin photodetector, graphene, Si_3_N_4_ membrane, thermopower

## Abstract

A broadband graphene-on-Si_3_N_4_-membrane photodetector for the visible-IR spectral range is realised by simple lithography and deposition techniques. Photo-current is produced upon illumination due to presence of the build-in potential between dissimilar metal electrodes on graphene as a result of charge transfer. The sensitivity of the photo-detector is ∼1.1 μA/W when irradiated with 515 and 1030 nm wavelengths; a smaller separation between the metal contacts favors gradient formation of the built-in electric field and increases the efficiency of charge separation. This optically-thin graphene-on-membrane photodetector and its interdigitated counterpart has the potential to be used within 3D optical elements, such as photonic crystals, sensors, and wearable electronics applications where there is a need to minimise optical losses introduced by the detector.

## 1. Introduction

Graphene—a two dimensional (2D) crystal—has become the subject of intense research for a variety of applications including optoelectronics due to its exceptional electronic [1,2,3], optical, physical and chemical properties [4,5,6]. Among the latest innovations, graphene has shown great potential in novel photonic devices such as photodetectors [1,2,3,4,6,7,8,9,10,11].

Despite the fact that graphene is a gapless material, it demonstrates strong interband absorption from a broad range of wavelengths between visible and near-infrared (near-IR). Graphene for use in photodetectors rely on one or more photo current generation principles. These are: photo-conductive, photo-thermoelectric (via Seebeck effect), and bolometric photo-voltage generation [4,12,13,14,15].

To decrease the response time of bolometric photodetectors, the use of materials/structures with a low thermal capacitance is required. Silicon nitride Si_3_N_4_ membranes are good candidates due to their low mass and excellent optical properties [16,17,18].

**Figure 1:**
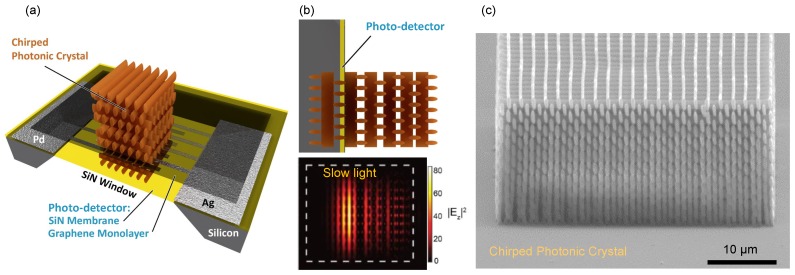
Photodetector concept for slow light monitoring inside a photonic crystal [19]. (**a**) Schematics of a graphene-based spectrally broadband photodetector for optical readout from within a spatially chirped photonic crystal. The photodetector has to be optically thin and non-perturbing to the localisation of slow light and consists of a nanomembrane of Si_3_N_4_, electrodes, and a graphene layer over the top (or below the contacts). (**b**) Side-view of the photodetector embedded inside the photonic crystal and modeling of light intensity inside photonic crystal at the slow light mode. It shows spatial localisation of the $E_{z}$ field component in the plane of the photodetector (calculations were carried without a simulated detector) [25,26]. (**c**) Electron-microscope image of photonic crystal with a spatial chirp (vertical) polymerised out of a negative-tone SZ2080 resist.

Motivation for this study is based on the current lack of photodetectors with small geometrical footprints which can be integrated into micro-/nano-resonant structures used in optical sensors [19]. In some applications, an ideal photodetector is a device that allows probing/monitoring light intensity without perturbing complex 3D photonic circuitry, e.g., slow light inside photonic crystals. Detection of slow light localization inside a 3D woodpile photonic crystal in the visible-IR range is shown in Figure 1 [20,21]. The E-field distribution of light in these structures can be calculated and examined indirectly, however, there are no tools to probe the field internally. Ideally, an optically thin detector has to be integrated inside 3D photonic lattices, e.g., made by state-of-the-art direct laser writing 3D nanolithography [22,23]. A photodetector which is weakly absorbing and has low dispersion (non-pertubing 3D photonic crystal) could be made using a graphene-on-membrane approach. Such thin sensors are currently not available and can become practical via cost effective manufacturing as explored in this study. Applications for such graphene photodetectors include flexible and wearable electronics, as well as applications where integration of multiple readout points for data analysis are required [24].

Here, we demonstrate a graphene-on-membrane photodetector using a micrometer thin Si_3_N_4_ membrane. Two different metals (Ag, Pd) are utilised as electrodes to create a build-in electric field across the graphene [12], between the electrodes. The bias due to charge carrier doping at the graphene-metal interface occurs as a result of differences in electron work function.

## 2. Experimental: Samples and Procedures

**Figure 2:**
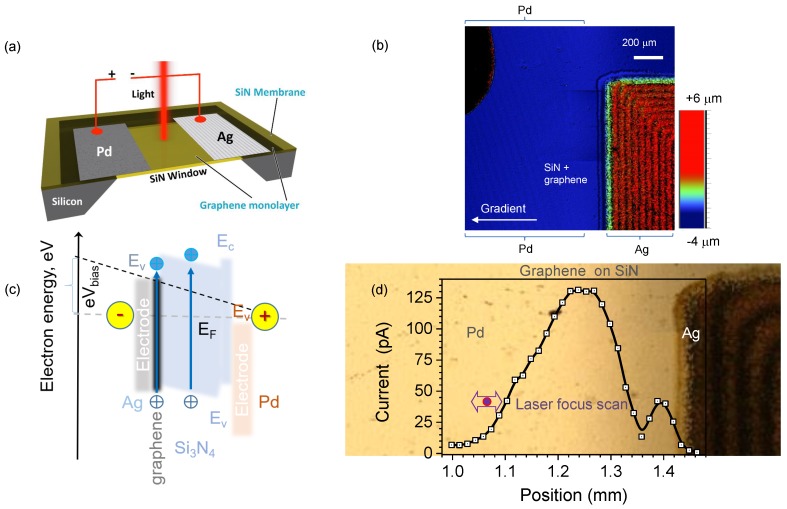
(**a**) Schematic illustration of the device: a monolayer of graphene sheet is placed on top of a Si_3_N_4_ membrane window, two metal pads (Pd and Ag) were then deposited on opposite sides across the Si_3_N_4_-window. Setup used for photodetector tests utilised a femtosecond laser operated at 200 kHz with a ∼230 fs pulse duration. Lock-in detection was carried out at 38 Hz. (**b**) Optical topography image of the photodetector. (**c**) Schematics of band alignment in the biased operation mode, e.g., photodetector (as in (**a**)) for n-type Si_3_N_4_ with graphene. Difference between electron work functions of Pd and Ag is ∼1 eV (Pd ∼5.5 eV, Ag ∼4.5 eV ). (**d**) The photo-current detected with lock-in amplifier by scanning the focal spot over the graphene surface on the Si_3_N_4_ membrane coated with Ag and Pd. Note, the Pd side has a gradient thickness due to shadowing of the cover glass used as a sputtering mask; Ag is deposited by a jet printer and had an edge region with edge-beads extending onto the graphene region. A photo is shown in the background of the plot to scale. Silver paste was used to attach Cu wire contacts. The Si_3_N_4_-window has a 0.5 mm square side-length.

In this work, the initial sample consists of a 7.5×7.5 mm${}^{2}$ Si substrate with a 1 μm thick Si_3_N_4_ film suspended over a 500×500 μm${}^{2}$ etched square in Si (Norcada, Ltd., Edmonton, AB, Canada) as shown in Figure 2a. A graphene sheet (ACS Material, Ltd., USA), estimated thickness 3.4 Å [27]) was transferred onto the substrate, centered over the Si_3_N_4_ window. Resistance of the graphene sheet (1 kΩ/sq) was measured by 4-point probe (Jandel Engineering Ltd., Leighton Buzzard, UK) and a UV-VIS-IR spectrometer scan showed an absorption value of 2.3% in the visible regime for one graphene monolayer. Furthermore, Raman spectroscopy was used to verify grapene’s transfer onto the substrate.

In the next step, a lithography-less approach was used to deposit the contacts. The first contact pad was deposited by using a 170-μm-thick microscope cover-slip as a shadow mask, leaving an open area on one side of the Si_3_N_4_ window to deposit a 100 nm thick Pd layer by magnetron sputtering (Axxis, KJ Lesker, Ltd., Jefferson Hills, PA, USA). The deposited layer had a linear thickness gradient over the detector window. The opposite contact was a silver (Ag) pad deposited by 3D jet printing (Aerosol Jet 300 Series Systems, Optomec, Ltd., USA) in 30-μm-wide lines and then annealed at 250 °C for 2 h. The final thickness of Ag was 1 μm. Conductivity σ=1/ρ of the Ag film was measured to be ∼20% of pure Ag, which is typical for silver pastes (the resistivity of silver is $\rho =1.59\times 10^{-8}$ Ωm). Importantly, this procedure was carried out on the 1 μm-thick membrane without any observable damage of membrane window. We note that both metals were deposited on the edges of Si_3_N_4_ membrane window (0.5×0.5 mm${}^{2}$) on top the graphene film. Figure 2a shows schematic diagram of the sample.

**Figure 3:**
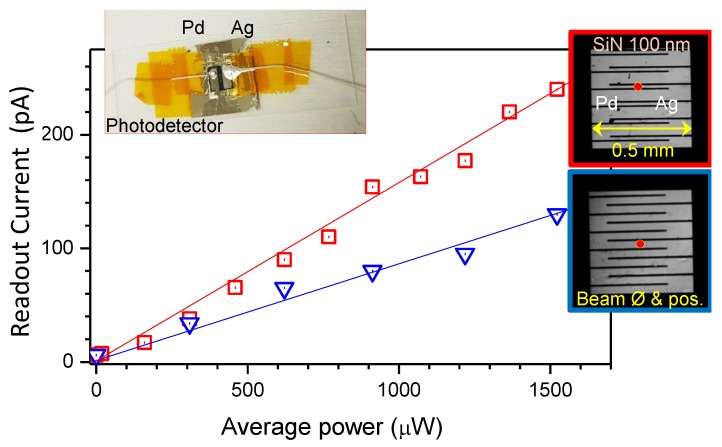
Response current of an IDE graphene photodetector for 1030 nm laser emission. The graphene layer was placed over Pd-Ag interdigitated electrodes made by two-step lithography with different distances between 8-μm-wide metallic contacts. Insets on the right show optical transmission images through a 100-nm-thick film of Si_3_N_4_ membrane. The inset shows the device used in experiments. Focal spot was ∼25 μm in diameter.

Another Si_3_N_4_ membrane photodetector with interdigitated electrodes (IDE) using Pd and Ag was fabricated as shown in Figure 3. The IDE design with 8 μm wide electrodes was written onto a 5×5×0.09 inch${}^{3}$ low reflective chromium-coated soda lime glass mask for photolithography (SF100 XPRESS, Intelligent Micropatterning Ltd.). Positive resist AZ1518 was spin coated on the substrate at 4000 rpm (thickness 1.8 μm) and exposed with the patterned mask centered over the SiN window. The resist was exposed under an i-line 365 nm UV lamp at 27 mW/cm${}^{2}$ for 1.8 s (total irradiation dose of 48.6 mJ/cm${}^{2}$) and then developed in AZ726 MIF for 20 s. In total, 100 nm of Pd was deposited by magnetron sputtering and then lifted-off in acetone to produce the first contact pad. The second 100 nm Ag contact pad was processed with the same lithography steps and deposited by electron beam evaporation (EBE). The graphene sheet was then transferred onto the SiN window and electrodes. Wires were soldered onto the electrodes to provide external electrical contact.

Photo-response of the graphene-Si_3_N_4_ membrane photodetector was confirmed using a 633 nm HeNe laser and linearity was characterised by femtosecond (fs)-laser with tunable pulse duration and repetition rate. A ∼230 fs pulse duration and 200 kHz pulse repetition rate fs-laser (Pharos, Light Conversion, Ltd., Lithuania) was used as a light source to heat up the Si_3_N_4_ membrane-graphene composite structure, with metal pads operating as the built in electrical bias (Figure 2a). The photo-response was measured by illuminating the membrane detector with a chopped laser beam at 1030 nm and 515 nm (second harmonic) wavelengths and recording the photo-current using a lock-in amplifier (SR530, Stanford Research Systems, Ltd., USA) via the $10^{6}$ V/A sensitivity port. The frequency of the lock-in amplifier locked by the chopper’s rotating frequency input was set to 38 Hz. The signal was detected by an oscilloscope (TDS 3054C Digital, Tektronix, Ltd.) and signal-to-noise ratio was increased using the lock-in amplifier frequency for synchronisation.

When both metal electrodes were of the same material, no photo-current was generated due to the same electric field between metal pads and the overall photo-response was zero as expected [10,14].

## 3. Results

### 3.1. Large Area Photodetector

The fs-laser beam was focused on the Si_3_N_4_-graphene membrane detector using an objective lens (Mitutoyo M Plan Apo 5×, numerical aperture NA=0.14) with the beam size of few microns in diameter d=1.22λ/NA, here λ is the wavelength of illumination (visible and near-IR excitation was used). The monolayer of graphene on Si_3_N_4_ membrane with metal electrodes deposited for current read-out comprises the photodetector with active sensor area of around 0.175 mm${}^{2}$. The aspect ratio photosensitive area is ∼7:9 and the total area of membrane between pads 0.2×0.5 mm${}^{2}$.

When the detector is irradiated, two mechanisms can lead to charge separation and photo-voltage or/and photo-current generation. The laser exposure causes a localised light absorbance and heating, resulting in a buildup of an electric field as photo-voltage via a photo-electric (Seebeck) mechanism [4]. Another mechanism is due to the electric field present between the two dissimilar metal electrodes, when photo-generated carriers are subjected to external bias and photo-current is generated upon illumination. These are the typical graphene photo-detector operation modes [7,8,11,12]. The photo-current mode of operation is more practical since generation of large temperature gradients near closely spaced (few micrometers) electrodes is challenging [4].

**Figure 4:**
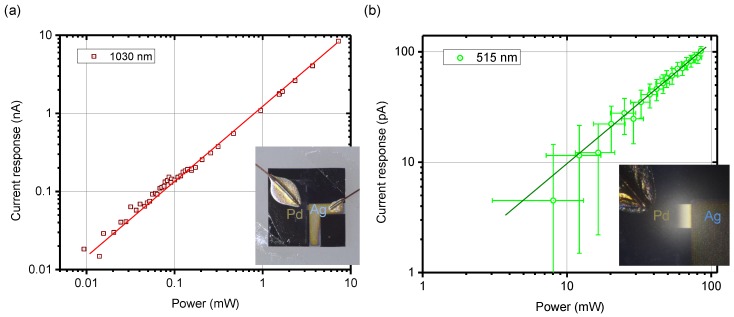
(**a**) Photodetector response vs. incident laser power at λ = 1030 nm; the largest measured value corresponds to the optical damage threshold of the upper surface (graphene side) of the sample. (**b**) Photodetector response vs. incident laser power at λ = 515 nm. Lines are eye guides for the linear slope γ=1. Insets show the same photodetector on the ∼7.5 × 7.5 ${\mathrm{mm}}^{2}$ Si chip (in (**a**)) and the close-up optical image on the central Si_3_N_4_-membrane window of 0.5×0.5 ${\mathrm{mm}}^{2}$ (in (**b**)). Averaging time per single data point was 10 s.

Next, a large area photodetector was tested. Laser illumination was directed onto the exposed ∼200 μm wide and a few millimeters long graphene sheet between the two metal electrodes (Pd and Ag). For the dissimilar metal electrodes, the built in electrical field caused charge separation and photo-current was detected as reported in ref. [14]. Figure 2d shows the measurement of photo-current at different locations on the photodetector carried out with a lock-in amplifier at λ=1030 nm and a laser spot diameter of ∼25 μm, which was small compared to the Si_3_N_4_ window in order to avoid reflections from the electrodes. A detailed view of the sensing region is shown by optical profilometry in Figure 2c. The optical reflection and transmission images of the active detector area and electrical contacts are shown in the insets of Figure 4 where a graded Pd coating is discernible. The largest photo-current was generated with the beam in the middle of the Si_3_N_4_ window (some edge irregularities close to the jet-printed Ag caused jumps in photo-current (Figure 2d)). When the laser beam was irradiating the metallic contacts with a stronger reflectivity, an overall reduction in total absorbed energy and photo-current readout was observed.

Experimental results of measured photo-current *vs.* laser power are shown in Figure 4. Linear response coefficients for the two wavelengths λ=1030 nm and 515 nm, as well as the corresponding sensitivities were $\chi_{1030}\approx 1.14\pm 0.05$ μA/W and $\chi_{515}\approx 1.18\pm 0.10$ μA/W, respectively. This was determined by linear best fit; for the input resistance of lock-in amplifier $10^{6}$ Ω. Correspondingly, the internal quantum efficiency (IQE) values estimated are ${\mathrm{IQE}}_{1030}=7\times 10^{-3}$% and ${\mathrm{IQE}}_{515}=14\times 10^{-3}$%. The IQE was calculated from the known absorbance of a single monolayer of graphene and photon flux (photons per second), i.e., incident photon conversion to electrons generated which are collected (the internal EQ). IQE=100% corresponds to every photon-generated charge carrier pair being collected at the electrodes.

The experimentally measured sensitivity values might appear lower than those of the most efficient photodetectors [7]. However, the responsivity values compare favorably with other types of graphene-on-Si_3_N_4_ photodetectors. The responsivity of the wave-guide enhanced configuration was reported to be ∼0.1 [28] and ∼0.32 mA/W [29] for the zero-biased operation. In addition, the design of the reported detector might allow it to detect light inside a 3D photonic crystal without perturbing transmission/reflection spectra due to its small form-factor.

Illumination of photodetector at the fourth harmonic at 257 nm wavelength was also tested. However, it was not possible to detect a change in current using irradiation doses less than the damage threshold for the Si_3_N_4_ membrane surface caused by strong absorption (NA=0.4 Mitutoyo near-UV objective lens optimised for high transmission at 257 nm). The transmission spectra of Si_3_N_4_ membrane confirmed a strong absorption at in the UV spectral range (not shown here). For around λ=1 μm (the thickness of the Si_3_N_4_ membrane d=2 μm), the expected value of the absorbtion coefficient is around α∼1.0 [${\mathrm{cm}}^{-1}$] [30]. Depending on the thickness, the Fabry–Perot interference in the SiN membrane significantly modulates transmission, which can be used to increase detection selectivity at specific wavelengths or to render the membrane transparent even at a high irradiance [31].

### 3.2. Photodetector for a Small Detection Area

The miniaturisation of the initial photodetector on a micro-Si_3_N_4_ membrane was tested earlier with a 4.5 μm gap width and dissimilar metal (Cr, Ag) electrodes [32] and was demonstrated on a bulk substrate [33].

Here, a more practical approach was taken, as opposed to high-resolution electron beam lithography [32]. The photodetector was defined by simple contact photolithography on a 100-nm-thick Si_3_N_4_-membrane and fabricated using magnetron sputtering of Pd and electron beam evaporation of Ag. The deposited layers were 35 nm thick. By alignment of the second pattern, the gap between the dissimilar metal electrodes could be made as required during the contact step (insets in Figure 3). Differently from the earlier described photodetector, the membrane was 10 times thinner and the graphene was transferred in the last step over the fabricated contacts. This further simplifies the fabrication of the thin photodetector. Slightly lower photo-sensitivity was observed (Figure 3) as compared with the device, where contacts were made over the graphene (Figure 4). A conformal graphene coating over the contacts and Si_3_N_4_-membrane was expected.

The partial transparency of the thin layer of Si_3_N_4_ in the visible and near-IR spectral ranges [30] is beneficial for transmission based optical devices. Specifically, 3D direct-laser-write fabrication processes [22] can be used for creating 3D structures on both sides of the photodetector. The benefit of such a photodetector could open up various multi-layer spatially integrable structures to be used in combination with thin-film photodetectors, which can operate in the biased as well as unbiased modes [34]; see the schematics in Figure 1a.

It is significant that a 100 nm thick Si_3_N_4_ membrane is resilient enough for multiple manual submersions in three different solvents over several fabrication steps. Therefore it is possible to use in complicated multi-step additive fabrication processes and benefit from mechanical properties of optically thin Si_3_N_4_ [35].

Future improvements are expected when a pyroelectric sub-micrometer-thin film of lithium niobate (or PZT) is placed below the graphene instead of the Si_3_N_4_ membrane. The pyroelectric layer generates bound charges under heating, which causes bandgap opening in graphene. It makes graphene function as a doped semiconductor. This feature can be utilised to allow detection by photo-voltage or photo-current [12]. The electron density induced in graphene is n=γ(T)T/e, where γ(T) is the temperature dependent pyroelectric coefficient, *e* is the electron charge, and *T* is the temperature increase [36]. Hence, optical absorption affects conductivity, which is a considerably stronger effect than the Seebeck mechanism, which generates low photo-voltage ΔV=S∇T even for the large Seebeck constant *S* of graphene [36]. The gradient thickness of metal contact used in this study can be also useful for measurement of the Seebeck constant of metal nanofilms, where it depends on the mean free path of electrons and phonon drag [37,38], and has to be experimentally determined for precise temperature measurements at the nanoscale [39].

## 4. Conclusions and Outlook

We demonstrate a photodetector made by lithography-free methods combining a simple graphene-to-Si_3_N_4_ membrane transfer method with jet-printing and magnetron sputtering for contact deposition. Sensitivity of the photodetector was ∼1.1 μA/W at both 1030 and 515 nm wavelengths when the Si_3_N_4_ membrane was 1 μm-thick. In addition, standard photolithography was used to define photodetector contacts on a 100-nm-thick Si_3_N_4_ membrane showing further miniaturization feasibility for visible and near-IR radiation detection at the nanoscale.

The two demonstrated modalities of photodetector fabrication via (1) lithography-free and direct-write of metal electrodes and (2) two-step photolithography on sub-micrometer-thick membranes opens new possibilities for the miniaturisation and integration of photodetectors. The new capability to embed such spectrally broad photodetectors into integrated systems, including those that can be 3D laser printed, is a promising direction (Figure 1). The sensitivity of such low-mass and transparent detectors need to be low so it is barely “visible” for the slow light (multiple reflections and interference) inside 3D photonic crystals.

The proposed photodetector was inferior to the current mature silicon photodetector technology in terms of sensitivity as it was not designed for maximum absorbance. However, for the particular function of detecting light inside 3D photonic structures without perturbation, the low sensitivity (invisibility) is the required property.

The proposed simple fabrication of photodetectors with sub-wavelength thickness can find applications in wearable opto-electronics and monitoring sensor applications.
